# Host sex, size, and hemoparasite infection influence the effects of ectoparasitic burdens on free‐ranging iguanas

**DOI:** 10.1002/ece3.4887

**Published:** 2019-01-15

**Authors:** Charles R. Knapp, Caro Perez‐Heydrich, Trevor T. Zachariah, Jill Jollay, Amy N. Schnelle, Sandra D. Buckner, Christine R. Lattin, L. Michael Romero

**Affiliations:** ^1^ Daniel P. Haerther Center for Conservation and Research John G. Shedd Aquarium Chicago Illinois; ^2^ Department of Biological Sciences Meredith College Raleigh North Carolina; ^3^ Brevard Zoo Melbourne Florida; ^4^ International Iguana Foundation Fort Worth Texas; ^5^ Veterinary Diagnostic Laboratory University of Illinois Urbana‐Champaign Illinois; ^6^ Villa Capulet Montague Foreshore Nassau The Bahamas; ^7^ Department of Biology Tufts University Medford Massachusetts; ^8^ Present address: Department of Biological Sciences Louisiana State University Baton Rouge Louisiana

**Keywords:** *Amblyomma*, body condition, *Cyclura cychlura*, health effects, parasite, ticks

## Abstract

Investigations focusing on host–ectoparasite interactions in animals have revealed asymptomatic to severe health and fitness consequences suggesting that species mobilize different interspecific response mechanisms. Fewer studies, however, have examined intraspecific responses to ectoparasitic burdens. In this study, we analyzed host health and fitness responses to increasing ectoparasite burdens along with the presence/absence of hemoparasites of free‐ranging insular rock iguanas (*Cyclura cychlura*) in The Bahamas. Using hematology, plasma biochemistry, as well as body condition and growth rate comparisons, we failed to find significant associations of tick burdens with annual growth rate, corticosterone, packed cell volume, total white blood cell, and heterophil, monocyte, eosinophil or hemoglobin measures. We did, however, find mixed and significant associations of tick burdens with lymphocyte and basophil counts, heterophil‐to‐lymphocyte ratios, and body condition indices. These associations varied by sex, size, and hemoparasite infection status suggesting that different life stages of iguanas may invest differently in immune responses, and impacts may be modulated based on size and sex of hosts, and coinfection status.

## 1 INTRODUCTION

Parasites are widespread in natural ecosystems and, by definition, infer some cost to their hosts (Watson, 2013). Indeed, parasites can hinder growth and reproduction (Fitze, Tschirren, & Richner, 2004; Uller & Olsson, 2004) and reduce long‐term survival in their hosts (Hair, Hoch, Buckner, & Barker, 1992; Jones et al., 2016; Madsen, Ujvari, & Olsson, 2005). Parasitic infestation can also affect biochemical and hematological values in their hosts (Quillfeldt, Masello, & Möstl, 2004; Raouf, Smith, Brown, Wingfield, & Brown, 2006; Wanless, Barton, & Harris, 1997). As such, host life histories are often mediated by parasites (Bull & Burzacott, 2006; Fitze et al., 2004; Michalakis & Hochberg, 1994) and are the outcomes of manifold adaptive behavioral responses to reduce the harmful effects of parasitism (Bui, Oppedal, Samsing, & Dempster, 2017; Moore, 2002).

To avoid costly effects of parasitism, hosts can mount a range of general defense strategies broadly including avoidance, resistance, and tolerance (Råberg, Graham, & Read, 2009). Owing to the high cumulative fitness costs of parasitism, there is strong selection for parasite avoidance—relying on indirect cues driven by long‐term associations—to minimize infection risk (Weinstein, Buck, & Young, 2018). Resistance and tolerance are host traits that have evolved to alleviate the health and fitness effects of parasitism. In general, resistance is a physiological response by the host to reduce parasitic burdens (including immunological resistance), while tolerance is the ability of a host to reduce the negative effect of infection on fitness at a set parasitic burden (Råberg et al., 2009). Each of these strategies could be influenced by predators (Marino, Holland, & Middlemis Maher, 2014), climate (Jones et al., 2016), or host traits (Comas, Ribas, Milazzo, Sperone, & Tripepi, 2014). For instance, levels of risk in hosts of different ages and body sizes will vary due to differences in their susceptibility to infection and in their age‐acquired immunity (Holland et al., 2007; Tinsley et al., 2012). Ultimately, host health and fitness may depend not only on the ability of a host to limit parasite burdens (avoidance and resistance) but also the damage caused by a given parasite burden (tolerance).

In parasitized hosts, ectoparasitic ticks are often assumed to confer a cost because when in large numbers adult ticks can consume a potentially significant amount of blood (Bull & Burzacott, 1993) and act as vectors for pathogens (Dunlap & Mathies, 1993). Investigations focusing on host–ectoparasite interactions in lizards, however, have revealed asymptomatic to severe health and fitness consequences (Bull & Burzacott, 1993; Jones et al., 2016; Main & Bull, 2000; Sorci & Clobert, 1995) suggesting that there are different interspecific response mechanisms in reptiles. For example, Galápagos marine iguanas (*Amblyrhynchus cristatus*) modify sleep locations, presumably as an ectoparasite avoidance strategy (Wikelski, 1999). Ectoparasite resistance in sand lizards (*Lacerta agilis*) has been linked to genetics (Olsson et al., 2005) and higher immunocompetence in side‐blotched lizards (*Uta stansburiana*; Spence, Durso, Smith, Skinner, & French, 2017), while tolerance has been documented in other lizard species (Bull & Burzacott, 1993; Gutsche, Mutschmann, Streich, & Kampen, 2012; Rivas, 2008). Fewer studies, however, have examined in detail the intraspecific responses to ectoparasitic burdens in lizards (Bull & Burzacott, 1993).

Blood‐borne apicomplexan endoparasites (hereafter referred as hemoparasites) are also found in many reptiles (Spence et al., 2017) and can have variable effects on their hosts. Brown, Shilton, and Shine (2006) failed to find empirical links between hemoparasite numbers and measures of fitness in keelback snakes (*Tropidonophis mairii*) including body condition, growth and feeding rates, and reproductive output. In Lilford's wall lizards (*Podarcis lilfordi*), however, there is a significant negative correlation between intensity of hemoparasite infection and burst speed (Garrido & Pérez‐Mellado, 2014). Iguanas (*Conolophus* spp.) with high hemoparasite loads from the Galápagos show significant alterations in heterophil‐to‐lymphocyte ratios (Onorati et al., 2017), while hemoparasites are uncorrelated with survival in the common lizard, (*Lacerta vivipara*; Sorci, Clobert, & Michalakis, 1996). In some reptile species, immunocompetence (i.e., bacterial killing ability) is only higher in individuals infected with both hemoparasites and ectoparasites (Spence et al., 2017).

Resistance protects the host at the expense of the parasite, while tolerance mitigates host costs without negatively affecting the parasite. However, hosts that are good at reducing parasite burdens may not necessarily appear the healthiest, while hosts with high parasite burdens can at times appear healthy (Råberg, 2014). For example, Jackson et al. (2014) documented an increase in body condition and enhanced survival in male rodents infected with the most parasites. An important metric to quantify the potential costs of parasitism on animal hosts is the difference in health and fitness parameters in relation to varying parasite burden, and how this relationship may be modulated by interactions with other parasites (Råberg et al., 2009). Reptiles in the wider Caribbean are parasitized by a unique community of acarine ectoparasites (Durden, Knapp, Beati, & Dold, 2015) and provide an interesting opportunity to study these host–parasite relationships under different but natural burden scenarios. In this study, we analyze host health and fitness responses to increasing ectoparasite burdens along with the presence/absence of hemoparasites of free‐ranging Exuma Island rock iguanas, *Cyclura cychlura figginsi* (Barbour, 1923) in The Bahamas, using hematology, plasma biochemistry, as well as body condition and growth rate comparisons.

The health condition of animals is often assessed using widely accepted veterinary tools including hematology and plasma biochemistry screening (Stacy, Alleman, & Sayler, 2011). Indeed, there is growing evidence regarding hematological effects of parasites in birds (Norte et al., 2013; Wanless et al., 1997), but relatively little work has been done to investigate similar host–parasite interactions in reptiles (Jacobson, 2007). We tested the hypothesis that parasites affect health of rock iguanas by analyzing changes in hematological characters (i.e., mean packed cell volume, hemoglobin, total white blood cell counts, absolute leukocyte counts, and heterophil‐to‐lymphocyte ratios) with increasing tick burdens.

Glucocorticoids are also often used to measure health in wild animals. Corticosterone is the primary adrenal glucocorticoid hormone produced in reptiles and birds to promote responses against stressful events (Hanke & Kloas, 1995), including ectoparasitic infestation (Raouf et al., 2006). If ticks act as chronic stressors, however, prolonged elevated corticosterone concentrations can disrupt normal physiological functions in reptiles (Guillette, Cree, & Rooney, 1995) and lead to stress‐related disease and pathology (Romero, Dickens, & Cyr, 2009). Thus, we tested the hypothesis that high parasite load is associated with elevated glucocorticoid levels (Maier & Watkins, 1999).

Ectoparasites and associated host blood loss can lead to regenerative anemia, resulting in changes in energy use, with energy being channeled into regeneration and self‐maintenance rather than growth (Bull & Burzacott, 1993). This could lead to decreased host fitness because clutch size is related to body size in *C. cychlura* (Knapp, Iverson, & Owens, 2006). The few studies addressing growth consequences of ectoparasite infestation suggest inconsistent responses of increased ectoparasite burden in lizards (Uller & Olsson, 2004; Cox & John‐Adler, 2007; Jessop et al., 2010). Likewise, most studies demonstrate a nonsignificant effect on body condition with increased ectoparasite burden (e.g., Brennan, Censky, & Powell, 2009; Spence et al., 2017; but see Klukowski & Nelson, 2001) suggesting that the precise energetic mechanisms for these effects are not clear (Cox & John‐Adler, 2007). Based on previous studies, we tested the hypothesis that there would be no growth or body condition costs associated with intensity of tick parasitism.

## 2 MATERIALS AND METHODS

### 2.1 Ethic statement

We minimized animal manipulations, handling times, and stress, in accordance with Shedd Aquarium veterinary and research committee guidelines, and with approval from The Bahamas Environmental, Science and Technology Commission. Samples were exported and imported under CITES permits issued to CRK.

### 2.2 Focal species and study sites

**Figure 1:**
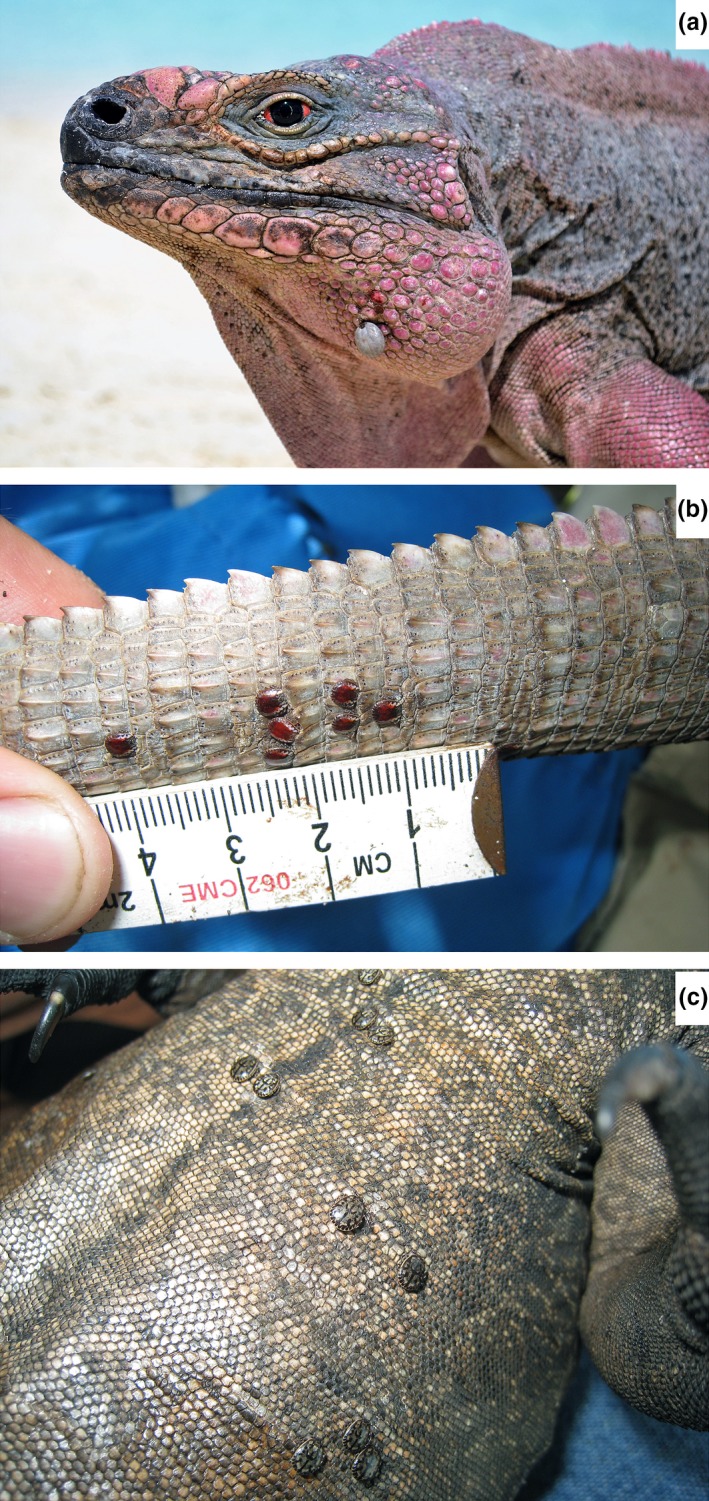
Blood‐engorged female tick (*Amblyomma torrei*) attached to a male host Exuma Island Rock Iguana (*Cyclura cychlura figginsi*) from the southern Exuma Cays (a); male ticks (*A. torrei*) from the southern Exuma Cays (b); and male ticks (*A. albopictum*) from the central Exuma Cays (c) in The Bahamas. Photograph credits Spencer Hudson (a) and Charles Knapp (b and c)

We conducted our study on *C. cychlura figginsi*, which is a large‐bodied (max. snout–vent length and body mass: 58.0 cm and 7.94 kg, respectively) herbivorous lizard, inhabiting isolated cays in the Exuma Islands chain, Bahamas. We conducted research on White Bay (4.6 ha), Noddy (5.9 ha), and North Adderly (5.9 ha) Cays in the southern Exuma Islands. The cays are characterized by low plant species richness, and similar diversity (Knapp et al., 2013). See Knapp and Alvarez‐Clare (2016) for description of representative vegetation communities for iguana‐inhabited cays. The tick species recorded from these southern Exuma Islands is *Amblyomma torrei* (Figure 1a). We also collected limited metrics (tick burden and body condition) from iguanas inhabiting Gaulin (13.2 ha) and Bitter Guana (73.5 ha) Cays in the central Exuma Islands. The tick species recorded from these two cays is *A. albopictum* (Figure 1b; Durden et al., 2015).

### 2.3 Corticosterone and hematology

From 28 to 31 March 2010, we captured iguanas from the three cays in the southern Exumas (White Bay [*N* = 40], Noddy [*N* = 33], and North Adderly [*N* = 43] Cays) using fish‐landing nets and nooses. Immediately following iguana capture, we collected blood samples by venipuncture of the ventral coccygeal vein using a heparinized syringe and stored them in vacutainer tubes containing sodium heparin. We collected all samples within 3 min of capture because corticosterone concentrations, the predominant glucocorticoid present in reptiles (reviewed by Moore & Jessop, 2003), start to increase 3 min after the initiation of an acute stressful stimulus in iguanas (Romero & Reed, 2005). After initial capture and blood sampling, we analyzed a 0.1 ml subsample of blood immediately in the field using a VetScan i‐STAT blood gas analyzer (Abaxis, Union City, CA, USA) with CG8+ cartridges to record hemoglobin.

We stored vacutainer tubes on ice until returning to the portable laboratory aboard our research vessel, where samples were processed within 12 hr of collection. We centrifuged a portion of each sample to harvest and immediately freeze plasma. We sent frozen aliquots to Tufts University (MA, USA) for corticosterone analyses by radioimmunoassay after extraction with dichloromethane (Wingfield, Vleck, & Moore, 1992) with an intra‐assay variability of 8%. The remainder of each blood sample was used to perform hematological evaluations. A small amount was placed into a microhematocrit tube and centrifuged to measure packed cell volume (PCV) using standard methodology (Voigt & Swist, 2011). We prepared two blood films per iguana by mixing an aliquot of blood with a 22% bovine serum albumin (BSA) solution (Sigma‐Aldrich, Inc., St. Louis, Missouri, USA) at a ratio of one drop BSA to five drops of blood. An additional amount of blood was stained using a phloxine B solution (Avian Leukopet kit; Vetlab Supply, Inc., Palmetto Bay, Florida, USA) and read for total white blood cells (WBC) using the Neubauer hemocytometer method (Campbell, 2015).

One of us (ANS) fixed and stained the blood films with Wright‐Giemsa at the University of Illinois College of Veterinary Medicine and reviewed each slide. We recorded the presence or absence of hemoparasites for each blood film from each animal but did not confirm taxonomy, though the cytologic appearance of the hemoparasites was compatible with *Haemogregarina* spp. Most organisms were oblong in shape, filled nearly or approximately half of the erythrocyte cytoplasm, and curved around the erythrocyte nucleus, with little or no nuclear displacement. Lower numbers of organisms were rounded and of variable diameter. We also performed a 100‐cell differential leukocyte count on the two blood films prepared from each study animal. We then averaged the two differential counts and combined the mean with data from the hemocytometer using a standard formula (Campbell, 2015), to calculate total leukocyte count and absolute numbers of each leukocyte type (cells/µl). We used absolute numbers for each cell type in our analyses as recommended by Stockham and Scott (2008). We also calculated the heterophil‐to‐lymphocyte ratio (H:L), which increases in response to stress in other reptiles (Mader, 2000).

### 2.4 Body condition and growth

We measured snout–vent length (SVL), tail length (TL), and body mass (BM) of each iguana and sexed by cloacal probing for hemipenes. During processing of each iguana, we counted ticks and recorded them as male, female, or as nymph/larvae. We calculated body condition indices (Stevenson & Woods, 2006) for each individual as the BM (in g)/SVL (in cm^3^). In other studies, lizards with high tick burdens 1 year tended to have high burdens the next year (Bull & Burzacott, 1993). To test for similar results, we performed paired *t* tests on tick burdens at first and last capture and found no significant difference (*t* = 0.139, *df* = 34, *p* = 0.890). We also calculated growth rates from 23 iguanas from the southern Exumas (White Bay, Noddy, and North Adderly Cays) recaptured during subsequent March study seasons. The number of days between recaptures ranged from 747 to 2,275 days (mean = 1692 days). Growth rate was estimated as the difference in SVL between the first and last captures, divided by the number of days separating the two captures.

### 2.5 Statistical analysis

To isolate the potential effects of feeding ticks, we considered tick burdens using both the number of attached ticks of all stages on the host (total tick burden) and as the burden of only ticks that feed on their hosts (adult females, larvae, nymphs; feeding tick burden). We used Spearman rank correlation coefficients to describe correlations between SVL and both total and feeding tick burdens. We used two sample *t* tests and analyses of variances (ANOVA) to compare mean total and feeding tick burdens between sexes and across cays, respectively. We further analyzed significant ANOVAs using pairwise tests of means, with *p*‐values adjusted for inflated type I error rates using the false discovery rate approach (Benjamini & Hochberg, 1995).

We restricted our regression analyses to only adult iguanas (SVL ≥ 26 cm) because smaller (i.e., younger) iguanas experience faster growth rates. Of the 116 adult iguanas captured, 65 provided complete data on hemoparasite infection. Thus, sample sizes for regression analyses ranged from 60 to 65 individuals, depending on the completeness of data on respective outcome variables. No individuals from Bitter Guana or Gaulin Cays were included in regression analyses because hemoparasite data were not collected from these sample populations. Additionally, growth rate data were available for 23 adults.

We fit multiple regression models addressing the relationship between individual hematological and physiological variables (summarized in Table 1), body condition index (BCI), and growth rates with total and feeding tick burdens to data from 65 adult iguanas from Noddy (*n* = 21), North Adderly (*n* = 21), and White Bay Cays (*n* = 23). Our regression models controlled for SVL, cay, and sex as potential confounders of the association between outcome variables and tick burdens. We included interaction terms in our full models that accounted for a varying effect of tick burden on hematological parameters based on iguana sex, size, and cay. We also included an interaction term that accounted for the differential effect of tick burdens based on whether an individual tested positive for a hemoparasite infection. In total, we fit thirteen multiple regression models of the following form to the data: *y* ~ Cay + Sex + SVL + Hemoparasite + Tick Burden + Cay:Tick Burden + Sex:Tick Burden + SVL:Tick Burden + Hemoparasite:Tick Burden, where *y* represents one of the 13 hematological/physiological variables (i.e., hematocrit, hemoglobin and packed cell volume values, body condition index, heterophil:lymphocyte ratios, white blood cell, heterophil, lymphocyte, monocyte, eosinophil, and basophil counts, corticosterone concentrations, and annual growth rates). We acknowledge that different distributional assumptions apply to different hematological/physiological outcome variables. Therefore, we used separate multiple linear regressions to model hematocrit, hemoglobin, and packed cell volume values, along with body condition index and heterophil:lymphocyte ratios, respectively. We used negative binomial regressions to model outcome variables associated with cell count data (i.e., white blood cell, heterophil, lymphocyte, monocyte, eosinophil, and basophil counts), and log‐normal regression models to model corticosterone and growth rate data, respectively.

**Table 1:** Summary statistics for iguana hematological parameters. Ninety‐five percent confidence intervals are listed parenthetically beside corresponding mean values. Sample sizes ranged from 40 to 43 for males and 20 to 22 for females because of differences in numbers of subjects with complete data. Sample size for growth rates includes 13 males and 10 females

	Adult males	Adult females
Corticosterone (ng/ml)	4.5 (3.0–6.0)	5.9 (3.7–8.1)
Average PCV (%)	26.0 (24.6–27.3)	25.3 (23.2–27.4)
Hematocrit value (%PCV)	21.7 (20.5–23.0)	20.4 (19.2–21.6)
WBCs (10^3^ cells/μl)	7.1 (5.9–8.2)	6.9 (5.1–8.7)
Heterophils (10^3^ cells/μl)	2.0 (1.6–2.4)	1.9 (1.2–2.6)
Lymphocytes (10^3^ cells/μl)	2.4 (1.8–3.0)	1.8 (1.3–2.3)
Monocytes (10^3^ cells/μl)	1.3 (1.0–1.5)	1.4 (0.9–2.0)
Eosinophils (10^3^ cells/μl)	0.8 (0.5–1.0)	1.1 (0.6–1.7)
Basophils (10^3^ cells/μl)	0.7 (0.5–0.9)	0.7 (0.4–0.9)
Hemoglobin (g/dl)	7.4 (7.0–7.8)	6.9 (6.5–7.3)
Heterophil:Lymphocyte ratio	1.2 (0.9–1.5)	1.1 (0.8–1.4)
Body condition index (10^−2^)	4.3 (4.1–4.4)	4.3 (4.2–4.4)
Growth rate (10^−3^ mm/day)	1.8 (1.2–2.4)	0.8 (0.3–1.4)

We plotted residuals of the fitted regression models for each of the hematological and physiological variables to evaluate compliance with regression model assumptions. Depending on whether a linear model or generalized linear model was used, we evaluated *F*‐statistics or chi‐square statistics associated with tick burden using type III ANOVA on fitted models. Based on fitted regression models for each outcome variable that were significantly associated with tick burden, we then calculated predicted (i.e., least squares) means across observed tick burden levels. These predicted means were adjusted for the effects of cay, sex, SVL, and interactions therein. When SVL interaction terms were significantly associated with the outcome variable, predicted means across tick burden levels were calculated at three SVL values based on sample tertiles (small = 27.8 cm; moderate = 31.1 cm; large = 35.8 cm). We conducted all statistical analyses in R 3.4.0 (R Core Development Team, 2017).

## 3 RESULTS

### 3.1 Total tick burden

#### 3.1.1 Summary statistics

**Figure 2:**
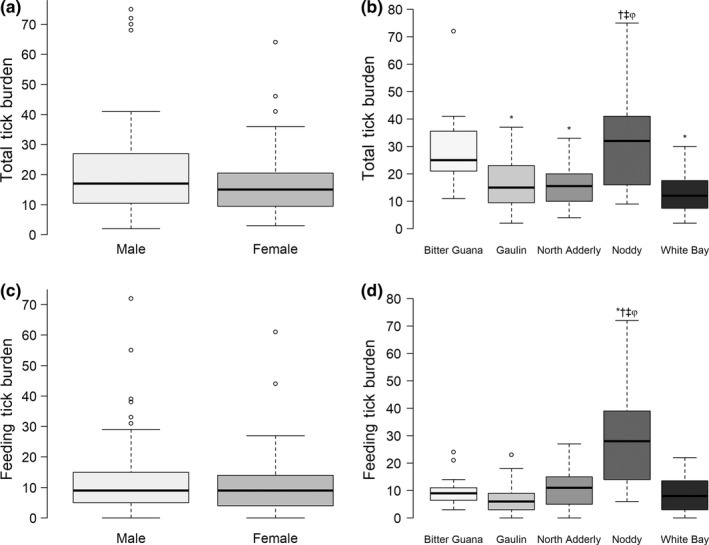
Bivariate associations between tick burden and covariates: SVL, sex, cay, and hemoparasite infection status. Mean total and feeding tick burden estimates from 43 adult males and 22 adult females are presented in panels (a) and (c), respectively. Panels (b) and (d) represent the distributions of total and feeding tick burdens, respectively, from iguanas captured at each cay during the sampling period (*n_Bitter Guana_* = 15, *n_Gaulin_* = 36, *n_North Adderly_* = 24, *n_Noddy_* = 21, *n_White_* *_Bay_* = 35,). Symbols on panels (b) and (d) correspond to significant differences in mean tick burden among individuals from focal cays and (*) Bitter Guana, (†) Gaulin, (‡) North Adderly, and (φ) White Bay Cays, after adjusting for inflated type I errors using the false discovery rate approach

Total tick burden was positively correlated with SVL (*r* = 0.465, *p* < 0.001). Mean total tick burdens did not significantly differ between males and females (males: 20.8 ticks, 95% CI: 17.6–30.0, range = 2–75; females: 17.1 ticks, 95% CI: 13.7–20.6, range = 3–64; *p* = 0.125; Figure 2a). Bitter Guana and Noddy Cay had significantly higher mean burdens of ectoparasitism relative to other sites (Bitter Guana: 29.7, 95% CI: 22.4—37.0; Noddy: 33.8, 95% CI: 24.9—42.7; Figure 2b). Sixty‐five adults provided data on hemoparasite infection, with overall prevalence of hemoparasitized hosts estimated at 55% (95% CI: 43–68%). Mean tick burden did not differ significantly between individuals with or without hemoparasite infection (infected: 21.2 ticks, 95% CI: 15.3–27.0; uninfected: 19.7 ticks, 95% CI: 14.8–24.6; *p* = 0.712).

#### 3.1.2 Regression analyses

After accounting for cay and interactions with sex, size, and presence of hemoparasites, total tick burden was found to be significantly associated with increases in BCI (*F*_1,53_ = 8.03, *p* = 0.006), increases in lymphocyte count ($\chi_{1}^{2}$ = 5.15, *p* = 0.023), increases in basophil count ($\chi_{1}^{2}$ = 5.30, *p* = 0.021), and decreases in H:L ratio (*F*_1,51_ = 5.97, *p* = 0.018). No significant associations were found between total tick burden and annual growth rate, corticosterone ($\chi_{1}^{2}$ = 1.56, *p* = 0.212), packed cell volume (*F*_1,61_ = 0.215, *p* = 0.645), total white blood cell ($\chi_{1}^{2}$ = 1.28, *p* = 0.258), heterophils ($\chi_{1}^{2}$ = 0.013, *p* = 0.910), monocytes ($\chi_{1}^{2}$ = 0.083, *p* = 0.773), eosinophils ($\chi_{1}^{2}$ = 0.081, *p* = 0.775), or hemoglobin values (*F*_1,56_ = 0.011, *p* = 0.918).

**Figure 3:**
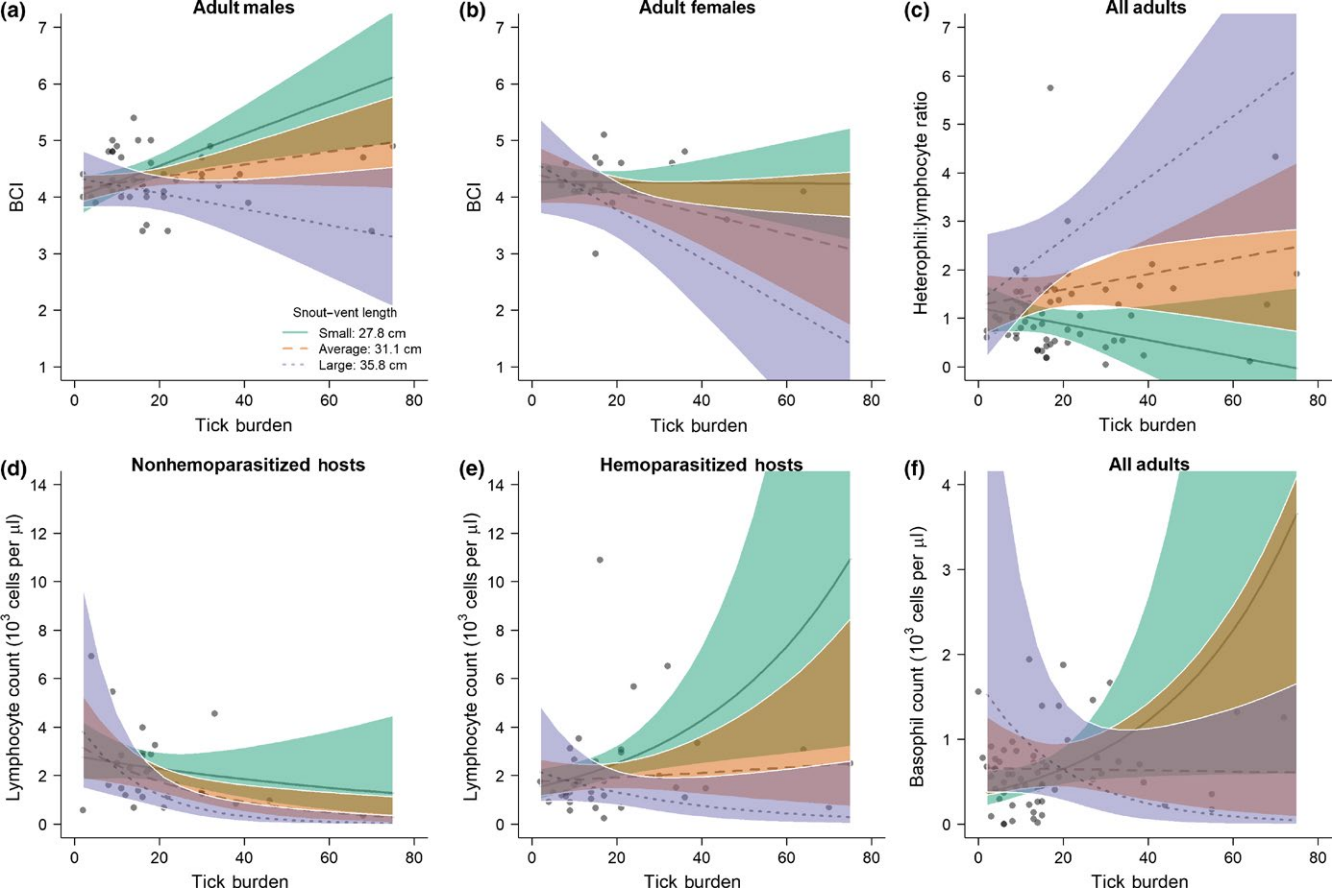
Predicted means of hematological and physiological variables that were significantly associated with total tick burden. Panels represent predicted means stratified by significant modifiers of tick burden effects (i.e., SVL and/or sex or hemoparasite infection status). Lines represent predicted means (and 95% confidence bands) as a function of tick burden and SVL, using three values of SVL based on sample tertiles (solid line: 27.8 cm; dashed line: 31.1 cm; dotted line: 35.8 cm)

There was a significant interaction between mean BCI and SVL (*F*_1,53_ = 7.35, *p = *0.009) and mean BCI and sex (*F*_1,53_ = 6.03, *p = *0.017). For both sexes, BCIs of larger individuals (SVL = 35.8 cm) declined with increases in total tick burden (Figure 3a,b); however, BCIs of smaller males (SVL = 27.8 cm and 31.1 cm) increased with tick burden (Figure 3a). Predicted mean BCIs of female iguanas declined with total tick burden, though the extent of this decline varied by SVL and was most notable for large females (Figure 3b). A significant interaction between total tick burden and SVL was detected for H:L ratios (*F*_1,51_ = 4.33, *p* = 0.012), whereby predicted mean ratios of larger individuals increased with tick burden, but decreased for smaller individuals (Figure 3c). Additionally, interactions between total tick burden and SVL ($\chi_{1}^{2}$ = 6.20, *p = *0.013), and total tick burden and hemoparasite infection ($\chi_{1}^{2}$ = 11.22, *p = *0.001) were significantly associated with lymphocyte count. For iguanas uninfected with hemoparasites, mean lymphocyte counts, adjusted for covariates, declined with increasing total tick burden regardless of SVL (Figure 3d). Among hemoparasite‐infected iguanas, lymphocyte counts increased as a function of total tick burden among smaller‐sized individuals (SVL ≤ 31.1 cm), but declined for larger individuals (Figure 3e). Predicted mean basophil counts increased with total tick burden for smaller‐sized individuals, but decreased for larger individuals (Figure 3f).

### 3.2 Feeding tick burden

#### 3.2.1 Summary statistics

Results associated with feeding tick burdens generally mirrored those of total tick burdens with few exceptions (Figure 2). Feeding tick burden was not significantly correlated with SVL (*r* = 0.157, *p* = 0.074). There was, however, a significant linear correlation between SVL and number of male ticks (i.e., total ticks − feeding ticks) detected on individual iguanas (*r* = 0.577, *p *= <0.001). Additionally, only Noddy Cay was significantly different from other sites with respect to mean feeding tick burdens (29.9 ticks, 95% CI: 26.0–33.8, Figure 2d). Mean feeding tick burden did not differ significantly between individuals with or without hemoparasite infection (infected: 17.0 ticks, 95% CI: 11.4–22.5; uninfected: 15.4 ticks, 95% CI: 11.0–19.9; *p* = 0.677).

#### 3.2.2 Regression analyses

After accounting for cay and interactions with sex, size, and hemoparasite infection, feeding tick burden was found to be significantly associated with overall increases in BCI (*F*_1,53_ = 6.22, *p* = 0.016), increases in basophil count ($\chi_{1}^{2}$ = 5.94, *p* = 0.015), increases in lymphocyte count ($\chi_{1}^{2}$ = 3.87, *p = *0.049), and decreases in H:L ratio (*F*_1,51_ = 5.02, *p* = 0.029); however, the direction and magnitude of these effects were often moderated by size, sex, and hemoparasitism. Significant interactions were found between BCI and SVL ($\chi_{1}^{2}$ = 5.29, *p = *0.025) and sex ($\chi_{1}^{2}$ = 5.51, *p = *0.023). For both sexes, BCIs of larger individuals (SVL = 35.8 cm) declined with increases in feeding tick burden (Supporting Information Figure S1a,b); however, BCIs of smaller males (SVL ≤ 31.1 cm) increased with feeding tick burden (Supporting Information Figure S1a). Predicted mean BCIs of females primarily declined with feeding tick burden, though the rate of decline was most pronounced for large females (Supporting Information Figure S1b). A significant interaction between feeding tick burden and SVL was also detected for H:L ratio (*F*_1,51_ = 5.76, *p* = 0.020), whereby predicted mean ratios of larger individuals increased with tick burden, but decreased for smaller individuals (Supporting Information Figure S1c). Effects of tick burden on lymphocyte counts were also moderated by SVL ($\chi_{1}^{2}$ = 4.55, *p = *0.033), as well as hemoparasitism ($\chi_{1}^{2}$ = 11.44, *p = *0.001), with predicted means of nonhemoparasitized hosts declining with feeding tick burden across all sizes, but increasing for smaller sizes among hemoparasitized hosts (Supporting Information Figure S1d,e). Predicted mean basophil counts increased with feeding tick burden for small‐sized adults, but declined in adults with SVL ≥ 31.1 cm (Supporting Information Figure S1f).

## 4 DISCUSSION

### 4.1 Sex and island differences in tick burdens

In this study, we failed to find significant associations of tick burdens with annual growth rate, corticosterone, packed cell volume, total white blood cell, and heterophil, monocyte, eosinophil or hemoglobin measures. We did, however, find mixed and significant associations of tick burdens with lymphocyte and basophil counts, H:L ratios, and body condition indices. These associations varied by sex, size, and hemoparasite infection rate supporting the supposition that different life stages of animals may invest differently in immune responses (Jackson et al., 2014; Soulsbury, Siitari, & Lebigre, 2018), or impacts may be modulated based on size and sex of hosts.

Our results demonstrate that total tick burdens are positively associated with SVL; however, there is no association with SVL when accounting for only ticks that consume a blood meal from their hosts (nymphs, larvae, adult females), suggesting that adult male ticks are driving the positive relationship between SVL and total tick burden. This relationship may be driven by competition among male ticks for space because ticks tend to aggregate in preferential attachment sites on lizard hosts (Dudek et al., 2016). Thus, larger iguanas may have more surface area at preferential attachment sites for male ticks. Temperature stability in larger iguana hosts may also play a role as ticks in the genus *Amblyomma* prefer a relatively narrow, warm band of host temperature for peak mating activity (Chilton & Andrews, 1987). There was no significant difference in mean tick burden between male and female host iguanas suggesting that these ticks lack an ability to differentiate between host sex (Pollock, Vredevoe, & Taylor, 2012).

Studies have reported elevated ectoparasitic infestation in higher‐density lizard populations (Bull, 1978; Smith, 1996). We suspect that the significantly elevated total tick burdens for iguanas from Noddy and Bitter Guana Cays may also be driven by higher population densities. Noddy Cay supports the highest density of iguanas (>45 iguanas/ha) from an undisturbed population (C. R. Knapp, unpublished data), while iguanas from Bitter Guana Cay congregate in high densities (100 iguanas/ha) on beaches to be fed by tourists. The proximity of individuals may facilitate the transfer of ectoparasites and is supported by evidence that these iguanas experience a greater incidence of endoparasitic infection at higher‐density sites (Knapp et al., 2013).

### 4.2 Hematological parameters and hemoparasitism

Though the specific functions of inflammatory cells remain poorly investigated in reptiles, increases or decreases in absolute counts of the leukocyte line may support clinical suspicion of inflammation, possibly in response to macroparasitic infection (Campbell, 2015). Indeed, wild tuatara (*Sphenodon punctatus*) experience an increase in total leukocytes (WBC) associated with tick burden (Burnham, Keall, Nelson, & Daugherty, 2006). However, these results are somewhat inconsistent, as other studies across different taxa have failed to find effects of ectoparasite burdens on leukocyte concentrations (Heylen & Matthysen, 2008; Sperry, Butler, Romero, & Weatherhead, 2008). After accounting for confounders, we found significant associations between tick burden and basophil counts, and lymphocyte counts, though the association between feeding tick burden and lymphocytes only approached significance. The association between tick burden and basophils, regardless of sex, size, or feeding category, increased as a function of tick burden, possibly as a first‐stage inflammatory response (histamine release) to tick attachment or tick‐borne pathogens (Jacobson, 2007).

Lymphocyte counts remained stable or declined as a function of total or feeding tick burden for all iguanas in our study except iguanas in the smallest size class (26.5 cm SVL) that were infected with hemoparasites (Figure 3e). Overall, immunological reactions to tick burden appeared to favor innate over adaptive immune responses for older individuals. Specifically, lymphocyte counts tended to decrease with tick burden (Figure 3d), whereas H:L ratios tended to increase with tick burden (Figure 3c). Immunosenescence (i.e., the decline in adaptive immune responses with age) has also been documented in other reptiles (Ujvari & Madsen, 2006). Differential patterns of immune investment could relate to trade‐offs associated with the energetic costs of innate vs. adaptive immune responses, and immune tolerance v. pathogen clearance responses throughout the lifetime of the host (Kutzer & Armitage, 2016; Rynkiewicz, Pederson, & Fenton, 2015). Increased inflammatory responses associated with ectoparasitism could have also contributed to decreases in BCI for females and older males. For adult females, the energetic costs of clearing infection associated with tick attachment may outweigh the benefits given the additional physiological demands associated with reproduction (Rynkiewicz et al., 2015). McDade, Georgiev, and Kuzawa (2016) hypothesized that low nutritional resource availability, low coinfection burden, and high mortality risk could bias investment toward innate immunity; however, it is difficult to determine whether our data fully support this claim. Stronger adaptive immune responses were observed among younger iguanas, especially when hemoparasite coinfections were present (Figure 3c,e). This is consistent with the findings from a previous study that also observed stronger adaptive immune responses in reptiles coinfected with hemoparasites and ectoparasites (Spence et al., 2017).

### 4.3 Corticosterone and heterophil‐to‐lymphocyte ratios

Previous studies evaluating the effects of tick burden on glucocorticoid levels of lizards have yielded conflicting results. For example, as in this study, tick burden did not correlate with corticosterone levels in side‐blotched lizards (*U. stansburiana*; Spence et al., 2017) or in two iguana species, *Conolophus marthae* and *C. subcristatus* (Onorati et al., 2017). However, Hanley and Stamps (2002) have reported a negative correlation between the intensity of ectoparasite burden and glucocorticoid plasma concentrations in black spiny‐tailed iguanas (*Ctenosaura similis*). The actual effect of tick burden on corticosterone concentrations most likely depends on multiple factors such as reproductive status and food limitations, thus requiring seasonal sampling. For example, ectoparasite burden was only positively related to corticosterone early in the reproductive season, at the time of ornamental color formation in sand lizards, *Lacerta agilis *(Lindsay, Wapstra, Silverin, & Olsson, 2016).

We did detect a significant association between tick burdens and H:L ratio, which has been argued to be a more representative and accurate measure of stress responses in wildlife (Davis, Maney, & Maerz, 2008; Romero & Reed, 2005). Additionally, we did find an overall correlation with H:L and corticosterone levels using all 65 iguanas in the study (Supporting Information Figure S2) as inferred from other studies (reviewed in Davis et al., 2008). One challenge in interpreting leukocyte profiles in wildlife is distinguishing between responses associated with inflammation or disease and stress responses associated with high‐energy expenditures (Davis et al., 2008). For example in birds, H:L ratios can increase in response to parasitic infection (Davis, Cook, & Altizer, 2004; Lobato, Moreno, Merino, Sanz, & Arriero, 2005) and the stress of long‐distance migration (Owen & Moore, 2006). Our models predicted notable increases in H:L ratios with tick burden for larger iguana hosts, while these ratios remained stable in medium‐sized iguanas and decreased in smaller iguanas. All iguanas in this study were sexually mature and presumably subject to similar stressors associated with mating, reproduction, nest construction, and defense (Knapp & Owens, 2005, 2008; Webb, Iverson, Knapp, DeNardo, & French, 2018) supporting the supposition that higher H:L ratios with increasing tick burdens in larger iguanas are an immunological response associated with increased energy expenditures in certain hosts (Sheldon & Verhulst, 1996).

### 4.4 Growth and body condition

Contrary to our prediction, body condition was negatively associated with tick burden for females and large males. Interestingly, trends in plots corresponding to H:L ratios were essentially the inverse (or opposite) of those depicted in plots corresponding to body condition indices (Figure 3c,d). Larger iguanas demonstrated the highest predicted mean H:L ratios and also the most pronounced declines in BCI as a function of tick burden. We suspect that these relationships are related to stress associated with greater energy expenditure for mate seeking, territory defense, and/or reproduction (Davis et al., 2008). Interestingly, BCI appeared to increase as a function of tick burden for small‐ and moderate‐sized males. Smaller‐ and medium‐sized male iguanas may be unconstrained from territorial defense and roam larger areas in search of food. Smaller males may also illicit minimal responses from larger territorial males, further allowing them to roam freely. Larger activity areas may potentially expose these males to more ticks, or infected conspecifics, but simultaneously provide opportunities to eat more calories and access to seasonal, higher‐quality diets. Though growth rates remained unchanged as a function of tick burden (see below), this supposition warrants further investigation by expanding the size ranges and animals under study and investigating size‐specific home ranges.

The few studies that have used growth as a testable effect of parasite burdens have focused on smaller species with shorter life spans, while recording growth over days to weeks (Uller & Olsson, 2004; Cox & John‐Adler, 2007). We recorded growth rates over a mean of 1692 days and failed to find significant evidence that tick infestation negatively affects growth. It may be that these lizards are tolerant of ectoparasitic infestation and that they may not impose growth costs (e.g., Spence et al., 2017; Uller & Olsson, 2004; but see Klukowski & Nelson, 2001). Alternatively, it may be that tick burdens in our study were below any threshold to induce fitness costs related to growth (Jessop et al., 2010).

### 4.5 Future considerations

We did not investigate reproductive output within our suite of physiological processes. When individuals are exposed to stressors such as parasites, more energy is needed than is typically available, resulting in changes to resource allocation among competing physiological processes including reproduction (Lucas & French, 2012). Indeed, ectoparasitic burdens are known to negatively affect reproductive success of birds (Fitze et al., 2004). Even though other studies in short‐lived reptiles found a positive correlation between hemoparasite load and host reproductive effort (Sorci et al., 1996), we suggest expanding studies to include reproductive output as a response variable in both hemo‐ and ectoparasitic investigations. Resource reallocation may perhaps play a larger role in long‐lived reptiles where clutch size varies with body size, and when there are more opportunities to oviposit over a lifetime (Knapp et al., 2006). Additionally, several ectoparasites including bloodsucking arthropods act as vectors for pathogens (Heylen & Matthysen, 2008), which can result in sublethal impacts such as reduced social interaction, altered thermoregulatory behavior, and altered energy reserves in lizards (Paranjpe et al., 2014; Schall, 1982). Therefore, future investigations should explore the behavioral and energy reallocation consequences of these parasites.

## CONFLICT OF INTEREST

None declared.

## AUTHOR CONTRIBUTIONS

CRK conceived the idea for the study. CRK, TTZ, JJ, and SDB conducted the fieldwork and collected the data. CRK, CP, TTZ, ANS, CRL, and LMR processed samples and analyzed data. All authors participated in writing and editing the manuscript.

## Data Availability

No database from the manuscript has been made publicly available yet. Data are available upon request. We intend to archive data from the manuscript in Dryad Digital Repository.

## References

[ece34887-bib-0001] Benjamini, Y. , & Hochberg, Y. (1995). Controlling the false discovery rate: A practical and powerful approach to multiple testing. Journal of the Royal Statistical Society. Series B. Statistical Methodology, 57, 289–300.

[ece34887-bib-0002] Brennan, A. M. , Censky, E. J. , & Powell, R. (2009). Effect of chigger mite (Acari: Trombiculidae) infestations on *Ameiva* (Squamata: Teiidae) from the Anguilla bank. Contemporary Herpetology, 2009, 1–3.

[ece34887-bib-0003] Brown, G. P. , Shilton, C. M. , & Shine, R. (2006). Do parasites matter? Assessing the fitness consequences of haemogregarine infection in snakes. Canadian Journal of Zoology, 84, 668–676.

[ece34887-bib-0004] Bui, S. , Oppedal, F. , Samsing, F. , & Dempster, T. (2017). Behaviour in Atlantic salmon confers protection against an ectoparasite. Journal of Zoology, 304, 73–80.

[ece34887-bib-0005] Bull, C. M. (1978). Heterogeneity of resource utilization in a population of the Australian reptile tick, *Aponomma hydrosauri* (Denny). Ecological Entomology, 3, 171–179.

[ece34887-bib-0006] Bull, C. M. , & Burzacott, D. (1993). The impact of tick load on the fitness of their lizard hosts. Oecologia, 96, 415–419. 28313658 10.1007/BF00317513

[ece34887-bib-0007] Bull, C. M. , & Burzacott, D. (2006). The influence of parasites on the retention of long‐term partnerships in the Australian sleepy lizard, *Tiliqua rugosa* . Oecologia, 146, 675–680. 16133193 10.1007/s00442-005-0224-z

[ece34887-bib-0008] Burnham, D. K. , Keall, S. N. , Nelson, N. J. , & Daugherty, C. H. (2006). Effects of sampling date, gender, and tick burden on peripheral blood cells of captive and wild tuatara (*Sphenodon punctatus*). New Zealand Journal of Zoology, 33, 241–248.

[ece34887-bib-0009] Campbell, T. W. (2015). Exotic animal hematology and cytology (4th ed.). Ames, IA: John Wiley & Sons Inc.

[ece34887-bib-0010] Chilton, N. B. , & Andrews, R. H. (1987). Mating behavior and parapatry in two species of Australian reptile tick. Oecologia, 75, 146–152. 10.1007/BF00378828 28311848

[ece34887-bib-0011] Comas, M. , Ribas, A. , Milazzo, C. , Sperone, E. , & Tripepi, S. (2014). High levels of prevalence related to age and body condition: Host‐parasite interactions in a water frog *Pelophylax* kl Hispanicus. Acta Herpetologica, 9, 25–31.

[ece34887-bib-0012] Cox, R. M. , & John‐Adler, H. B. (2007). Increased mite parasitism as a cost of testosterone in male striped plateau lizards *Sceloporus virgatus* . Functional Ecology, 21, 327–334.

[ece34887-bib-0013] Davis, A. K. , Cook, C. K. , & Altizer, S. (2004). Leukocyte profiles of House Finches with and without mycoplasmal conjunctivitis, a recently emerged bacterial disease. EcoHealth, 1, 362–373.

[ece34887-bib-0014] Davis, A. K. , Maney, D. L. , & Maerz, J. C. (2008). The use of leukocyte profiles to measure stress in vertebrates: A review for ecologists. Functional Ecology, 22, 760–772.

[ece34887-bib-0015] Dudek, K. , Skórka, P. , Sajkowska, Z. A. , Ekner‐Grzyb, A. , Dudek, M. , & Tryjanowski, P. (2016). Distribution pattern and number of ticks on lizards. Ticks and Tick Borne Diseases, 7, 172–179. 26520053 10.1016/j.ttbdis.2015.10.014

[ece34887-bib-0016] Dunlap, K. D. , & Mathies, T. (1993). Effects of nymphal ticks and their interactions with malaria on the physiology of male fence lizards. Copeia, 1993, 1045–1048.

[ece34887-bib-0017] Durden, L. A. , Knapp, C. R. , Beati, L. , & Dold, S. (2015). Reptile‐associated ticks from Dominica and the Bahamas with notes on hyperparasitic erythraeid mites. Journal of Parasitology, 101, 24–27. 25274575 10.1645/14-602.1

[ece34887-bib-0018] Fitze, P. S. , Tschirren, B. , & Richner, H. (2004). Life history and fitness consequences of ectoparasites. Journal of Animal Ecology, 73, 216–226.

[ece34887-bib-0019] Garrido, M. , & Pérez‐Mellado, V. (2014). Sprint speed is related to blood parasites, but not to ectoparasites, in an insular population of lacertid lizards. Canadian Journal of Zoology, 92, 67–72.

[ece34887-bib-0020] Guillette, L. J. , Cree, A. , & Rooney, A. A. (1995). Biology of stress: Interactions with reproduction, immunology and intermediary metabolism In Warwick C., Frye F. L., & Murphy J. B. (Eds.), Health and welfare of captive reptiles (pp. 32–81). London, UK: Chapman & Hall.

[ece34887-bib-0021] Gutsche, A. , Mutschmann, F. , Streich, W. J. , & Kampen, H. (2012). Ectoparasites in the endangered Utila spiny‐tailed iguana (*Ctenosaura bakeri*). Herpetological Journal, 22, 157–161.

[ece34887-bib-0022] Hair, J. A. , Hoch, A. L. , Buckner, R. G. , & Barker, R. W. (1992). Fawn hematology and survival following tick infestation and Theileriasis. Journal of Agricultural Entomology, 9, 301–319.

[ece34887-bib-0023] Hanke, W. , & Kloas, W. (1995). Comparative aspects of regulation and function of the adrenal complex in different groups of vertebrates. Hormone and Metabolic Research, 27, 389–397. 8557236 10.1055/s-2007-979986

[ece34887-bib-0024] Hanley, K. A. , & Stamps, J. A. (2002). Does corticosterone mediate bidirectional interactions between social behaviour and blood parasites in the juvenile black iguana, *Ctenosaura similis*? Animal Behaviour, 63, 311–322.

[ece34887-bib-0025] Heylen, D. J. A. , & Matthysen, E. (2008). Effect of tick parasitism on the health status of a passerine bird. Functional Ecology, 22, 1099–1107.

[ece34887-bib-0026] Holland, M. P. , Skelly, D. K. , Kashgarian, M. , Bolden, S. R. , Harrison, L. M. , & Cappello, M. (2007). Echinostome infection in green frogs (*Rana clamitans*) is stage and age dependent. Journal of Zoology, 271, 455–462.

[ece34887-bib-0027] Jackson, J. A. , Hall, A. J. , Friberg, I. M. , Ralli, C. , Lowe, A. , Zawadzka, M. , … Begon, M. (2014). An immunological marker of tolerance to infection in wild rodents. PLoS Biology, 12(7), e1001901. 25004450 10.1371/journal.pbio.1001901 PMC4086718

[ece34887-bib-0028] Jacobson, E. R. (2007). Parasites and parasitic diseases of reptiles In Jacobson E. R. (Ed.), *Infectious diseases and pathology of reptiles*: *C**olor atlas and text* (571–666). Boca Raton, FL: CRC Press.

[ece34887-bib-0029] Jessop, T. S. , Summer, J. , Imansyah, J. , Purwandana, D. , Seno, A. , Ariefiandy, A. , & Ciofi, C. (2010). Assessment of environmental and host dependent factors correlated with tick abundance on Komodo dragons. Australian Zoologist, 35, 265–275.

[ece34887-bib-0030] Jones, A. R. , Bull, C. M. , Brook, B. W. , Wells, K. , Pollock, K. H. , & Fordham, D. A. (2016). Tick exposure and extreme climate events impact survival and threaten the persistence of a long‐lived lizard. Journal of Animal Ecology, 85, 598–610. 26559641 10.1111/1365-2656.12469

[ece34887-bib-0031] Klukowski, M. , & Nelson, C. E. (2001). Ectoparasite loads in free‐ranging Northern Fence Lizards, *Sceloporus undulates hyacinthinus*: Effects of testosterone and sex. Behavioral Ecology and Sociobiology, 49, 289–295.

[ece34887-bib-0032] Knapp, C. R. , & Alvarez‐Clare, S. (2016). Influence of morphological, chemical and physical leaf traits on food selection of a herbivorous iguana from The Bahamas. Journal of Tropical Ecology, 32, 75–78.

[ece34887-bib-0033] Knapp, C. R. , Iverson, J. , & Owens, A. K. (2006). Geographic variation in nesting behavior and reproductive biology of an insular iguana (*Cyclura cychlura*). Canadian Journal of Zoology, 84, 1566–1575.

[ece34887-bib-0034] Knapp, C. R. , & Owens, A. K. (2005). Home range and habitat associations of a Bahamian iguana: Implications for conservation. Animal Conservation, 8, 269–278.

[ece34887-bib-0035] Knapp, C. R. , & Owens, A. K. (2008). Nesting behavior and the use of termitaria by the Andros Iguana (*Cyclura cychlura cychlura*). Journal of Herpetology, 42, 46–53.

[ece34887-bib-0036] Knapp, C. R. , Hines, K. N. , Zachariah, T. , Perez‐Heydrich, C. , Iverson, J. B. , Buckner, S. D. , … Romero, L. M. (2013). Physiological effects of tourism and associated food provisioning in an endangered iguana. Conservation Physiology, 1, 1–12. 10.1093/conphys/cot032 PMC4806617 27293616

[ece34887-bib-0037] Kutzer, M. A. M. , & Armitage, S. A. O. (2016). Maximizing fitness in the face of parasites: A review of host tolerance. Zoology, 119, 281–289. 27373338 10.1016/j.zool.2016.05.011

[ece34887-bib-0038] Lindsay, W. R. , Wapstra, E. , Silverin, B. , & Olsson, M. (2016). Corticosterone: A costly mediator of signal honesty in sand lizards. Ecology and Evolution, 6, 7451–7461. 28725412 10.1002/ece3.2318 PMC5513280

[ece34887-bib-0039] Lobato, E. , Moreno, J. , Merino, S. , Sanz, J. J. , & Arriero, E. (2005). Haematological variables are good predictors of recruitment in nestling pied flycatchers (*Ficedula hypoleuca*). Ecoscience, 12, 27–34.

[ece34887-bib-0040] Lucas, L. D. , & French, S. S. (2012). Stress‐induced tradeoffs in a free‐living lizard across a variable landscape: Consequences for individuals and populations. PLoS ONE, 7, e49895. 23185478 10.1371/journal.pone.0049895 PMC3502225

[ece34887-bib-0041] Mader, D. R. (2000). Normal hematology of reptiles In Feldman B. F., Zinkl J. G., & Jain N. C. (Eds.), Schalm's veterinary hematology (5th ed., 1126–1132). Philadelphia, PA: Lippincott Williams & Wilkins.

[ece34887-bib-0042] Madsen, T. , Ujvari, B. , & Olsson, M. (2005). Old pythons stay fit; effects of haematozoan infections on life history traits of a large tropical predator. Oecologia, 142, 407–412. 15517406 10.1007/s00442-004-1742-9

[ece34887-bib-0043] Maier, S. F. , & Watkins, L. (1999). Bidirectional communication between the brain and the immune system: Implications for behaviour. Animal Behaviour, 57, 741–751.

[ece34887-bib-0044] Main, A. R. , & Bull, C. M. (2000). The impact of tick parasites on the behaviour of the lizard *Tiliqua rugosa* . Oecologia, 122, 574–581. 28308351 10.1007/s004420050981

[ece34887-bib-0045] Marino, J. A. Jr , Holland, M. P. , & Middlemis Maher, J. (2014). Predators and trematode parasites jointly affect larval anuran functional traits and corticosterone levels. Oikos, 123, 451–460.

[ece34887-bib-0046] McDade, T. W. , Georgiev, A. V. , & Kuzawa, C. W. (2016). Trade‐offs between acquired and innate immune defenses in humans. Evolution, Medicine, & Public Health, 2016, 1–16. 10.1093/emph/eov033 PMC4703052 26739325

[ece34887-bib-0047] Michalakis, Y. , & Hochberg, M. E. (1994). Parasitic effects on host life‐history traits: A review of recent studies. Parasite, 1, 291–294. 9140497 10.1051/parasite/1994014291

[ece34887-bib-0048] Moore, I. T. , & Jessop, T. S. (2003). Stress, reproduction, and adrenocortical modulation in amphibians and reptiles. Hormones and Behavior, 43, 39–47. 12614633 10.1016/s0018-506x(02)00038-7

[ece34887-bib-0049] Moore, J. (2002). Parasites and the behavior of animals. New York, NY: Oxford University Press Inc.

[ece34887-bib-0050] Norte, A. C. , Lobata, D. N. C. , Braga, E. , Antonini, Y. , Lacorte, G. , Goncalves, M. , … Ramos, J. A. (2013). Do ticks and *Borrelia burgdorferi* s. l. constitute a burden to birds? Parasitology Research, 112, 1903–1912. 23430359 10.1007/s00436-013-3343-1

[ece34887-bib-0051] Olsson, M. , Madsen, T. , Wapstra, E. , Silverin, B. , Ujvari, B. , & Wittzell, H. (2005). MHC, health, color, and reproductive success in sand lizards. Behavioral Ecology and Sociobiology, 58, 289–294.

[ece34887-bib-0052] Onorati, M. , Sancesario, G. , Pastore, D. , Bernardini, S. , Cruz, M. , Carrión, J. E. , … Gentile, G. (2017). Effects of parasitic infection and reproduction on corticosterone plasma levels in Galápagos land iguanas, *Conolophus marthae* and *C. subcristatus* . Ecology and Evolution, 7, 6046–6055. 28808564 10.1002/ece3.3077 PMC5551272

[ece34887-bib-0053] Owen, J. C. , & Moore, F. R. (2006). Seasonal differences in immunological condition of three species of thrushes. Condor, 108, 389–398.

[ece34887-bib-0054] Paranjpe, D. A. , Medina, D. , Nielsen, E. , Cooper, R. D. , Paranjpe, S. A. , & Sinervo, B. (2014). Does thermal ecology influence dynamics of side‐blotched lizards and their microparasites? Integrative and Comparative Biology, 54, 108–117. 24920752 10.1093/icb/icu069

[ece34887-bib-0055] Pollock, N. B. , Vredevoe, L. K. , & Taylor, E. N. (2012). How do host sex and reproductive state affect host preference and feeding duration of ticks? Parisitology Research, 111, 897–907. 10.1007/s00436-012-2916-8 22526292

[ece34887-bib-0056] Quillfeldt, P. , Masello, J. F. , & Möstl, E. (2004). Blood chemistry in relation to nutrition and ectoparasite load in Wilson's storm‐petrels *Oceanites oceanicus* . Polar Biology, 27, 168–176.

[ece34887-bib-0057] R Core Development Team . (2017). R: A language and environment for statistical computing. Vienna, Austria: R Foundation for Statistical Computing.

[ece34887-bib-0058] Råberg, L. (2014). How to live with the enemy: Understanding tolerance to parasites. PLoS Biology, 12(11), e1001989 10.1371/journal.pbio.1001989 25369060 PMC4219658

[ece34887-bib-0059] Råberg, L. , Graham, A. L. , & Read, A. F. (2009). Decomposing health: Tolerance and resistance to parasites in animals. Philosophical Transactions of the Royal Society of London. Series B, Biological Sciences, 364, 37–49. 18926971 10.1098/rstb.2008.0184 PMC2666700

[ece34887-bib-0060] Raouf, S. A. , Smith, L. C. , Brown, M. B. , Wingfield, J. C. , & Brown, C. R. (2006). Glucocorticoid hormone levels increase with group size and parasite load in cliff swallows. Animal Behaviour, 71, 39–48.

[ece34887-bib-0061] Rivas, J. A. (2008). Ticks (*Amblyomma* spp.) on black iguanas (*Ctenosaura similis*) in Costa Rica. Iguana, 15, 25–27.

[ece34887-bib-0062] Romero, L. M. , Dickens, M. J. , & Cyr, N. E. (2009). The reactive scope model‐a new model integrating homeostasis, allostasis, and stress. Hormones and Behavior, 55, 375–389. 19470371 10.1016/j.yhbeh.2008.12.009

[ece34887-bib-0063] Romero, L. M. , & Reed, J. M. (2005). Collecting baseline corticosterone samples in the field: Is under 3 min good enough? Comparative Biochemistry and Physiology Part A: Molecular and Integrative Physiology, 140, 73–79. 10.1016/j.cbpb.2004.11.004 15664315

[ece34887-bib-0064] Rynkiewicz, E. C. , Pederson, A. B. , & Fenton, A. (2015). An ecosystem approach to understanding and managing within‐host parasite community dynamics. Trends in Parasitology, 31, 212–221. 25814004 10.1016/j.pt.2015.02.005

[ece34887-bib-0065] Schall, J. J. (1982). Lizards infected with malaria: Physiological and behavioral consequences. Science, 217, 1057–1059. 7112113 10.1126/science.7112113

[ece34887-bib-0066] Sheldon, B. C. , & Verhulst, S. (1996). Ecological immunology: Costly parasite defences and trade‐offs in evolutionary ecology. Trends in Ecology and Evolution, 11, 317–321. 21237861 10.1016/0169-5347(96)10039-2

[ece34887-bib-0067] Smith, G. R. (1996). Mites on stripped Plateau lizards (*Sceloporus virgatus*: Phrynosomatidae): Abundance, distribution and effects on host growth. Herpetological Natural History, 4, 175–180.

[ece34887-bib-0068] Sorci, G. , & Clobert, J. (1995). Effects of maternal parasite load on offspring life‐history traits in the common lizard (*Lacerta vivipara*). Journal of Evolutionary Biology, 8, 711–723.

[ece34887-bib-0069] Sorci, G. , Clobert, J. , & Michalakis, Y. (1996). Cost of reproduction and cost of parasitism in the common lizard, *Lacerta vivipara* . Oikos, 76, 121–130.

[ece34887-bib-0070] Soulsbury, C. D. , Siitari, H. , & Lebigre, C. (2018). Stabilising selection on immune response in male black grouse *Lyrurus tetrix* . Oecologia, 186, 405–414. 29177843 10.1007/s00442-017-4014-1 PMC5799332

[ece34887-bib-0071] Spence, A. R. , Durso, A. M. , Smith, G. D. , Skinner, H. M. , & French, S. S. (2017). Physiological correlates of multiple parasitic infections in side‐blotched lizards. Physiological and Biochemical Zoology, 90, 321–327. 28384422 10.1086/691059

[ece34887-bib-0072] Sperry, J. H. , Butler, L. K. , Romero, L. M. , & Weatherhead, P. J. (2008). Effects of parasitic infection and radio‐transmitters on condition, hematological characteristics and corticosterone concentrations in Texas ratsnakes. Journal of Zoology, 278, 100–107.

[ece34887-bib-0073] Stacy, N. I. , Alleman, A. R. , & Sayler, K. A. (2011). Diagnostic hematology of reptiles. Clinics in Laboratory Medicine, 31, 87–108. 21295724 10.1016/j.cll.2010.10.006

[ece34887-bib-0074] Stevenson, R. D. , & Woods, W. A. (2006). Condition indices for conservation: New uses for evolving tools. Integrative and Comparative Biology, 46, 1169–1190. 21672816 10.1093/icb/icl052

[ece34887-bib-0075] Stockham, S. L. , & Scott, M. A. (2008). Fundamentals of veterinary clinical pathology (2nd ed). Carlton, Vic., Australia: Blackwell Publishing.

[ece34887-bib-0076] Tinsley, R. , Stott, L. , York, J. , Everard, A. , Chapple, S. , Jackson, J. , … Tinsley, M. C. (2012). Acquired immunity protects against helminth infection in a natural host population: Long‐term field and laboratory evidence. International Journal of Parasitology, 42, 931–938. 22906507 10.1016/j.ijpara.2012.07.006

[ece34887-bib-0077] Ujvari, B. , & Madsen, T. (2006). Age, parasites, and condition affect humoral immune response in tropical pythons. Behavioral Ecology, 17, 20–24.

[ece34887-bib-0078] Uller, T. , & Olsson, M. (2004). Ectoparasite susceptibility in lizard populations sympatric and allopatric with ticks. Ecoscience, 11, 428–432.

[ece34887-bib-0079] Voigt, G. L. , & Swist, S. L. (2011). Hematology techniques and concepts for veterinary technicians. Chichester, UK: John Wiley and Sons.

[ece34887-bib-0080] Wanless, S. , Barton, T. R. , & Harris, M. P. (1997). Blood hematocrit measurements of 4 species of North Atlantic seabirds in relation to levels of infestation by the tick *Ixodes uriae* . Colonial Waterbirds, 20, 540–544.

[ece34887-bib-0081] Watson, M. J. (2013). What drives population‐level effects of parasites? Meta‐analysis meets life history. International Journal for Parasitology: Parasites and Wildlife, 2, 190–196. 24533334 10.1016/j.ijppaw.2013.05.001 PMC3862538

[ece34887-bib-0082] Webb, A. C. , Iverson, J. B. , Knapp, C. R. , DeNardo, D. F. , & French, S. S. (2018). Energetic investment associated with vitellogenesis induces an oxidative cost of reproduction. Journal of Animal Ecology, 10.1111/1365-2656.12936 30521087

[ece34887-bib-0083] Weinstein, S. B. , Buck, J. C. , & Young, H. S. (2018). A landscape of disgust. Science, 359, 1213–1214. 29590062 10.1126/science.aas8694

[ece34887-bib-0084] Wikelski, M. (1999). Influences of parasites and thermoregulation on grouping tendencies in marine iguanas. Behavioral Ecology, 10, 22–29.

[ece34887-bib-0085] Wingfield, J. C. , Vleck, C. M. , & Moore, M. C. (1992). Seasonal changes of the adrenocortical response to stress in birds of the Sonoran Desert. Journal of Experimental Zoology, 264, 419–428. 1460439 10.1002/jez.1402640407

