# Supplementary figures and images for: Host sex, size, and hemoparasite infection influence the effects of ectoparasitic burdens on free‐ranging iguanas
Source: Ecol Evol. 2019 Jan 15;9(4):1946–56. doi: 10.1002/ece3.4887 (PMC6392384; doi:10.1002/ece3.4887)

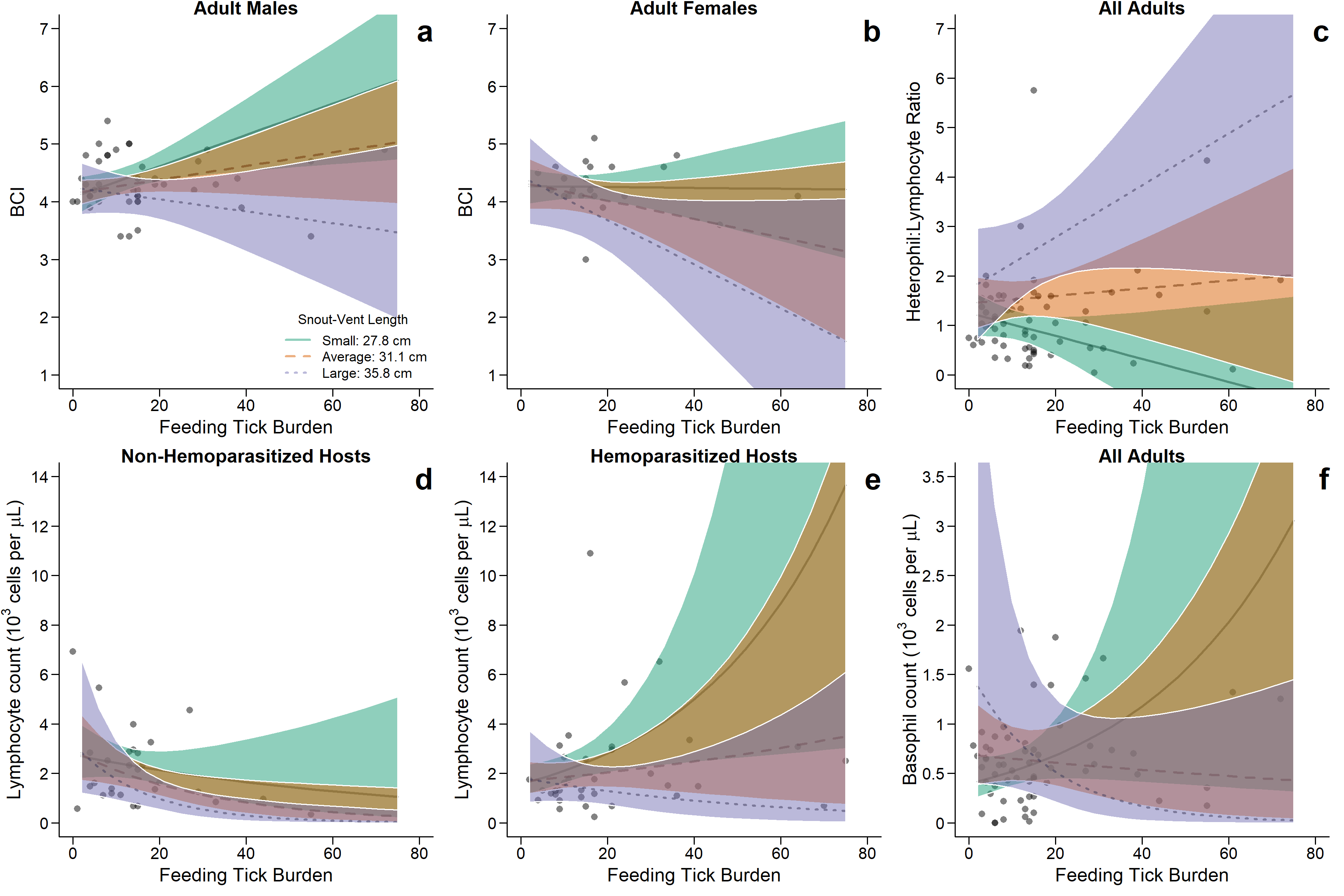

Supplement: Supplementary file 1 [file ECE3-9-1946-s001.tif]

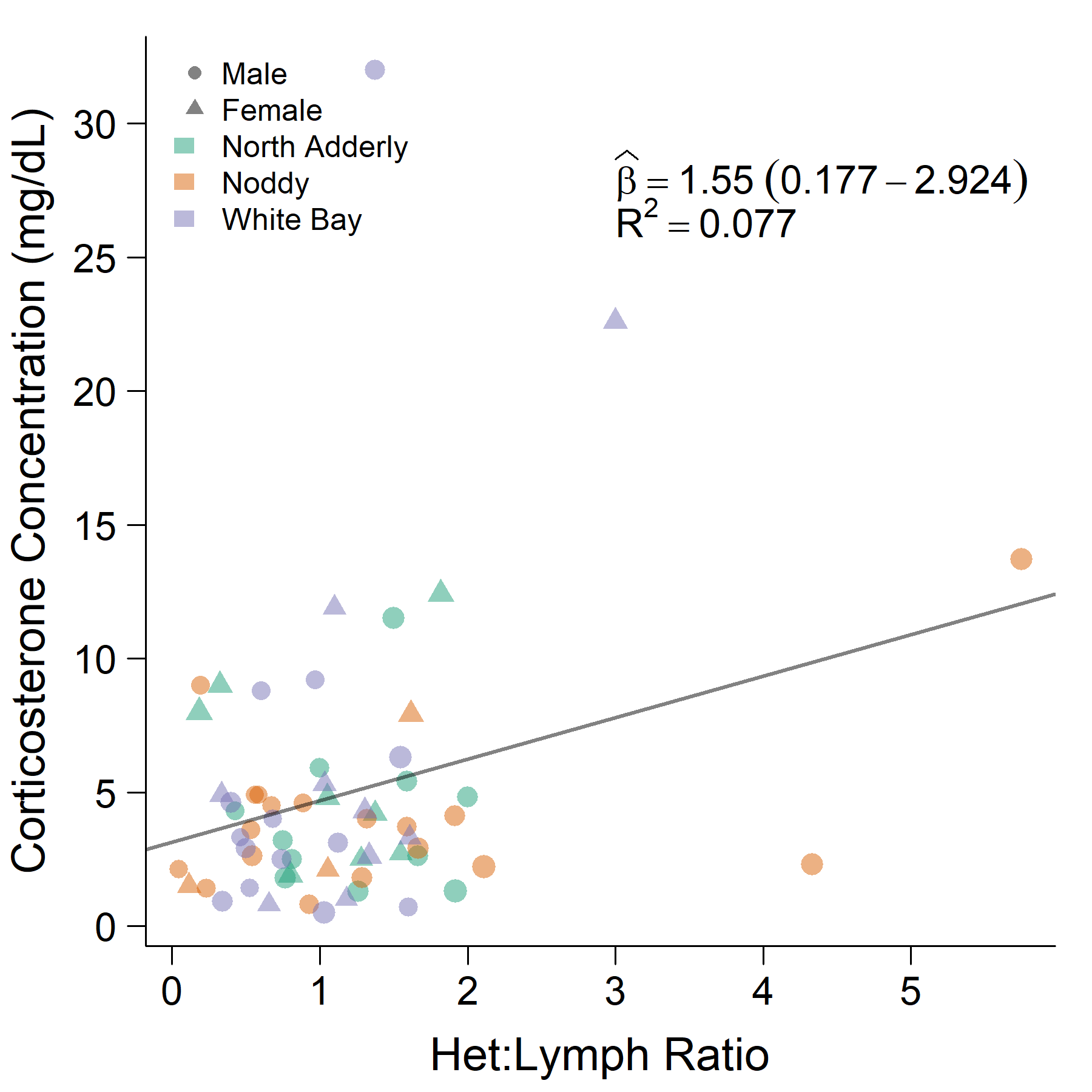

Supplement: Supplementary file 2 [file ECE3-9-1946-s002.tif]
